# Alpha-tocopherol attenuates the anti-tumor activity of crizotinib against cells transformed by NPM-ALK

**DOI:** 10.1371/journal.pone.0183003

**Published:** 2017-08-14

**Authors:** Yuki Uchihara, Fumihito Ueda, Kenji Tago, Yosuke Nakazawa, Tomoyuki Ohe, Tadahiko Mashino, Shigenobu Yokota, Tadashi Kasahara, Hiroomi Tamura, Megumi Funakoshi-Tago

**Affiliations:** 1 Division of Hygienic Chemistry, Faculty of Pharmacy, Keio University, Minato-ku, Tokyo, Japan; 2 Division of Structural Biochemistry, Department of Biochemistry, Jichi Medical University, Shimotsuke-shi, Tochigi-ken, Japan; 3 Division of Medicinal Chemistry and Bio-organic Chemistry, Faculty of Pharmacy, Keio University, Minato-ku, Tokyo, Japan; 4 International University of Health and Welfare, Graduate School, Minato-ku, Tokyo, Japan; Seoul National University College of Pharmacy, REPUBLIC OF KOREA

## Abstract

Anaplastic large cell lymphomas (ALCL) are mainly characterized by harboring the fusion protein nucleophosmin-anaplastic lymphoma kinase (NPM-ALK). The ALK inhibitor, crizotinib specifically induced apoptosis in Ba/F3 cells expressing NPM-ALK by inhibiting the activation of NPM-ALK and its downstream molecule, signal transducer and activator of transcription factor 3 (STAT3). We found that α-tocopherol, a major component of vitamin E, attenuated the effects of crizotinib independently of its anti-oxidant properties. Although α-tocopherol suppressed the inhibitory effects of crizotinib on the signaling axis including NPM-ALK and STAT3, it had no influence on the intake of crizotinib into cells. Crizotinib also directly inhibited the kinase activity of NPM-ALK; however, this inhibitory effect was not altered by the co-treatment with α-tocopherol. Whereas the nuclear localization of NPM-ALK was disappeared by the treatment with crizotinib, the co-treatment with α-tocopherol swept the effect of crizotinib and caused the localization of NPM-ALK in nucleus. The administration of α-tocopherol attenuated the anti-tumor activity of crizotinib against NPM-ALK-provoked tumorigenesis *in vivo*. Furthermore, the α-tocopherol-induced inhibition of crizotinib-caused apoptosis was also observed in NPM-ALK-positive cells derived from ALCL patients, namely, SUDHL-1 and Ki-JK. Collectively, these results not only revealed the novel mechanism underlying crizotinib-induced apoptosis in NPM-ALK-positive cells, but also suggest that the anti-tumor effects of crizotinib are attenuated when it is taken in combination with vitamin E.

## Introduction

Anaplastic lymphoma kinase (ALK) is a receptor-type protein tyrosine kinase that belongs to the insulin-receptor superfamily [1]. ALK shows high sequence similarity to receptor tyrosine kinases such as LTK and ROS1 [2,3]. Alterations in ALK gene including chromosomal translocations, mutations, and overexpression have been implicated in the development of ALK-positive human cancers including anaplastic large cell lymphomas (ALCL) and non-small cell lung cancers (NSCLC). Among the ALK-positive ALCL patients, approximately 80% harbor the t(2;5)(p23;q35) translocation [4], causing the expression of nucleophosmin-anaplastic lymphoma kinase (NPM-ALK). NPM-ALK includes N-terminal oligomerization domain of NPM and C-terminal kinase domain of ALK. By mediated N-terminal NPM region, the NPM-ALK automatically forms a dimer, and exhibits disordered activation, which is essential for cellular transformation [5]. NPM-ALK triggers the activation of various pro-survival signaling pathways such as signal transducer and activator of transcription factor 3 (STAT3) and extracellular signal-regulated kinase (ERK), which are involved in cell cycle progression, proliferation, survival, and oncogenesis [6,7]. In 2007, ALK was shown to fuse to echinoderm microtubule-associated protein-like 4 (EML4), yielding the fusion kinase EML4-ALK detected in approximately 3–5% of NSCLC. Similar to NPM-ALK, EML4-ALK constitutively formed dimers and exhibited oncogenic activity [8].

Crizotinib is an orally available ALK inhibitor that directly inhibits the kinase activity of ALK, and also suppresses receptor tyrosine kinases including c-Met and ROS1 [9,10]. Crizotinib was approved by the US Food and Drug Administration for the treatment of ALK-positive NSCLC in 2011 [10]. Previous case reports showed that crizotinib was effective in the treatment of ALCL [11,12]. The mechanisms by which crizotinib exhibits its anti-tumor activity against various tumors harboring the mutation in ALK genes currently remain unclear. A recent study reported that a treatment with crizotinib resulted in the accumulation of reactive oxygen species (ROS) in human alveolar rhabdomyosarcoma and embryonal rhabdomyosarcoma cells [13], suggesting that crizotinib exhibits anti-tumor activity through the generation of ROS.

In the present study, we attempted to clarify the mechanisms by which crizotinib exhibits its anti-tumor activity by investigating its effects on the proliferation and tumor formation by NPM-ALK. Interestingly, we found that α-tocopherol attenuated the anti-tumor activity of crizotinib independently of its anti-oxidant properties by performing *in vitro* and *in vivo* analysis. Our results not only clarified some of the mechanisms by which crizotinib exerts its anti-tumor effects, but also suggest that the intake of vitamin E attenuates the anti-tumor effects of crizotinib.

## Materials and methods

### Reagents

Recombinant murine IL-3 was purchased from PEPROTECH (Rocky Hill, NJ, USA). Puromycin was purchased from InVivoGen (San Diego, CA, USA). Crizotinib (PF-02341066; Xalkori) was presented by Pfizer (San Diego, CA, USA). Mitomycin C (MMC) were purchased from Kirin Brewery Co. (Tokyo, Japan). α-Tocopherol, δ-tocopherol and anti-Flag (M2) antibody were purchased from Sigma-Aldrich (St. Louis, MO, USA). β-Tocopherol and γ-tocopherol were purchased from Abcam (Cambridge, MA, USA). α-Tocotrienol and Trolox were purchased from Cayman Chemical (Ann Arbor, MI). Anti-phospho-STAT3 antibody (Tyr^705^), anti-phospho-STAT5 antibody (Tyr^694^) and anti-STAT5 antibody were purchased from Cell Signaling Technology (Danvers, MA, USA). Anti-β-actin antibody and anti-STAT3 antibody were purchased from Santa Cruz Biotechnology Inc. (Santa Cruz, CA). Peroxidase-conjugated rabbit anti-mouse and goat anti-rabbit secondary antibodies were from Dako (Glostrup, Denmark).

### Plasmids

The cDNA encoding NPM-ALK harboring Flag tag on its N terminus was inserted into the MSCV-Puro retroviral vector. The mutagenesis of amino acid residues in NPM-ALK (K210R) was performed using a site-directed mutagenesis kit according to the manufacturer’s instructions (Stratagene, La Jolla, CA, USA). MSCV-IRES-GFP-TEL-JAK2 was gifted by Dr. J.N. Ihle (St. Jude Children’s Research Hospital, Memphis, TN, USA).

### Retrovirus infection and cell culture

The IL-3-dependent hematopoietic cell lines Ba/F3 were infected with an empty virus (-) and retroviruses expressing NPM-ALK, its kinase dead mutant (K210R), and TEL-JAK2 using RetroNectin according to the manufacturer’s instructions (Takara Bio Inc., Shiga, Japan). These cells were cultured in RPMI-1640 medium (Nacalai Tesque, Tokyo, Japan) supplemented with 10% fetal bovine serum (FBS) (BioWest, Nuaillé, France), 100 units/ml penicillin (Nacalai Tesque), 100 μg/ml streptomycin (Nacalai Tesque), 2 ng/mL IL-3 (PEPROTECH), and 5 μg/mL puromycin (InVivoGen). SUDHL-1 and Ki-JK cells, derived from NPM-ALK-positive ALCL patients, were cultured in RPMI 1640 medium supplemented with 10% FBS (BioWest), 100 units/ml penicillin (Nacalai Tesque), and 100 μg/ml streptomycin (Nacalai Tesque) with or without IL-3 (2 ng/mL) at 37°C and 5% CO_2_.

### Cell culture and retrovirus infection

The IL-3-dependent hematopoietic cell line Ba/F3 was purchased from Riken Cell Bank (Ibaraki, Japan). SUDHL-1 and Ki-JK cells, derived from NPM-ALK-positive ALCL patients, were purchased from Summit Pharmaceuticals International (Tokyo, Japan) and JCRB cell Bank (Osaka, Japan), respectively. SUDHL-1 cells and Ki-JK cells were cultured in RPMI 1640 medium supplemented with 10% FBS (BioWest), 100 units/ml penicillin (Nacalai Tesque), and 100 μg/ml streptomycin (Nacalai Tesque) with or without IL-3 (2 ng/mL) at 37°C and 5% CO_2_. Ba/F3 cells were infected with an empty virus (-) and retroviruses expressing NPM-ALK, its kinase dead mutant (K210R), and TEL-JAK2 using RetroNectin according to the manufacturer’s instructions (Takara Bio Inc., Shiga, Japan). These cells were cultured in RPMI-1640 medium (Nacalai Tesque, Tokyo, Japan) supplemented with 10% fetal bovine serum (FBS) (BioWest, Nuaillé, France), 100 units/ml penicillin (Nacalai Tesque), 100 μg/ml streptomycin (Nacalai Tesque), 2 ng/mL IL-3 (PEPROTECH), and 5 μg/mL puromycin (InVivoGen).

### Measurement of intracellular ROS generation

The accumulation of intracellular ROS was detected using 2', 7'-dichlorodihydrofluorescein diacetate (DCFH-DA) (Cayman Chemical, Ann Arbor, MI, USA), which was hydrolyzed by a cellular esterase to 2', 7'-dichlorodihydrofluorescein (DCFH) and then oxidized to the fluorescent compound 2', 7'-dichlorofluorescein (DCF). Cells were incubated with PBS containing DCFH-DA (10 μM) at 37°C for 1 hr, and then washed with PBS. Cells were treated with crizotinib or pyrrolidinium fullerene in combination with α-tocopherol for 24 hr and washed with PBS. The fluorescence intensity of oxidized DCF was monitored using FACS Calibur with the CELL Quest program as previously described [14].

### Cell proliferation assay and measurement of cell viability

Transduced Ba/F3 cells (1×10^5^ cells/500 μL) were cultured with RPMI supplemented with 10% FBS, 100 units/ml penicillin, and 100 μg/ml streptomycin in a 24-well plate. After 24-hr and 48-hr incubations, living cells were counted using a Beckman Coulter Vi-Cell (Beckman Coulter, Fullerton, CA) by the Trypan blue exclusion method. In the water-soluble tetrazolium (WST) assay, transduced Ba/F3 cells (5×10^4^ cells/100 μL) were cultured in a 96-well plate in the presence of crizotinib and/or α-tocopherol, β-tocopherol, γ-tocopherol, α-tocotrienol, and Trolox. After 24 hr, 10 μL of Cell Count Reagent SF (Nacalai Tesque) was added to each well, and cells were incubated at 37°C in 5% CO_2_ for 2 hr. Absorbance was measured at 450/690 nm using the microplate reader, Infinite 200 PRO (Tecan, Switzerland). Cell viability was calculated by absorbance.

### Cell cycle analysis

Cells were fixed with 70% (v/v) ethanol at -20°C overnight. They were then centrifuged at 5,000 r.p.m at 4°C for 2 min and treated with PBS containing 10 μg/ml RNase A (Nacalai Tesque). After the addition of 100 μg/ml propidium iodide (PI) (Wako Pure Chemical Industries, Tokyo, Japan), cell cycle parameters were assessed by a flow cytometric analysis using FACS Calibur as described previously [14].

### DNA fragmentation assay

Genomic DNA was prepared for gel electrophoresis as described previously [15]. Electrophoresis was performed on a 1% (w/v) agarose gel in Tris/boric acid buffer. Fragmented DNA was visualized by staining with ethidium bromide after electrophoresis.

### Immunoblot analysis

Cells were washed with PBS and lysed in NP-40 lysis buffer (50 mM Tris-HCl pH 7.4, 10% glycerol, 50 mM NaCl, 0.5% sodium deoxycholate, 0.5% NP-40, 20 mM NaF, and 0.2 mM Na_3_VO_4_) supplemented with protease inhibitors. Cell lysates were cleared by centrifugation at 15,000 r.p.m at 4°C for 15 min and proteins were then denatured with Laemmli buffer. For preparation of cytosolic fraction and nuclear fraction, cells were disrupted in Buffer A (10 mM Hepes-KOH (pH7.8), 10 mM KCl, 0.1 mM EDTA (pH8.0), 0.1% NP-40) and were centrifuged at 5,000 r.p.m for 15 min at 4°C. The supernatant was collected and used as cytosolic fraction. Isolated nuclei were lysed in Nonidet P-40 lysis buffer and homogenized using the ultrasonic homogenizer VP-50 (TAITEC, Japan), and then centrifuged at 15,000 r.p.m at 4°C for 15 min in order to remove debris. Cytosolic fraction and nuclear fraction were denatured with Laemmli's sample buffer. Denatured proteins were resolved by SDS-PAGE and transferred onto PVDF membranes (Millipore, Billerica, MA). Membranes were probed using the designated antibodies and visualized with the ECL detection system (GE Healthcare UK., Ltd.). The intensity of each band was quantified by Image-J software. The phosphorylation levels of STAT3 and STAT5 were normalized with the expression levels of STAT3 and STAT5. To show the relative amounts of NPM-ALK in cytosol and nucleus, the band intensity of NPM-ALK were normalized with band intensities of β-tublin and Lamin B, respectively.

### Measurement of crizotinib uptake by LC-MS

Ba/F3 cells expressing NPM-ALK (9×10^4^ cells/200 μL) were cultured with crizotinib (0.5 μM) in combination with α-tocopherol (25 μM) for 60 or 120 min and washed with ice-cold PBS, and then lysed with 125 μL of 50% acetonitrile. Samples were centrifuged at 5,000 r.p.m at 4°C for 2 min and the supernatant was analyzed by an Agilent 6120 quadrupole mass spectrometer equipped with an electrospray interface attached to an Agilent 1200 series (Agilent Technologies, Santa Clara, CA, USA). Chromatographic separations were performed on Agilent ZORBAX Eclipse Plus C18 column (4.6 × 100 mm, 3.5 μm) at a flow rate 0.5 mL/min. The mobile phases used were water with 0.1% formic acid (A) and acetonitrile with 0.1% formic acid (B). At time zero the flow consisted of 90% of mobile phase A and 10% mobile phase B. One minute after injection, the proportion of B was linearly increased to 100% over 4 min followed by keeping constant at 100%B for 5 min. From this point, the mobile phase was set to initial conditions (90%A and 10%B) and the column was equilibrated for 5 min prior to the next injection. The electrospray ionization probe was set at 350°C, with the nebulizing gas pressure and electrospray gas flow set at 20 psi. and 13 L/min, respectively. Detection of crizotinib was carried out by selected ion monitoring of [M+H]^+^ ions at m/z 450 in the positive ion mode.

### *In vitro* kinase assay

NPM-ALK autophosphorylation was measured by modified methods as described previously [16]. Cells were lysed in NP-40 lysis buffer supplemented with protease inhibitors and NPM-ALK was immunoprecipitated using the anti-Flag antibody and protein G-sepharose (GE Healthcare Biosciences, Pittsburgh, PA, USA) at 4°C for 4 hr. Immunocomplexes were washed three times with NP-40 lysis buffer and twice with kinase buffer (20 mM Tris-HCl pH 7.4, 5 mM MgCl_2_, 5 mM MnCl_2_, and 1 mM dithiothreitol). The kinase reaction was performed in 30 μL of kinase buffer including 30 μM ATP (Cytoskeleton, Inc., Denver, CO, USA) and 10 μCi [γ-^32^P] ATP (PerkinElmer, Inc. Waltham, MA, USA) at 30°C for 15 min. The reaction was terminated by the addition of Laemmli buffer and further boiling for 10 min. Proteins were separated by SDS-PAGE and ^32^P-labeled proteins were measured by autoradiography. To show the relative phosphorylation level of NPM-ALK, the band intensity of phosphorylated NPM-ALK was normalized with expression level of NPM-ALK.

### Transplantation of tumor cells into nude mice

Six-week-old female BALB/c nude mice were subcutaneously injected with transduced Ba/F3 cells (2×10^7^ cells). In order to investigate oncogenic potentials *in vivo*, tumor, liver, and lymph node weights were analyzed 16 days after transplantation. Regarding the administration of crizotinib and α-tocopherol, nude mice were randomized into three groups; vehicle, crizotinib, and crizotinib plus α-tocopherol. Crizotinib (1 mg) and/or α-tocopherol (4 mg) were dissolved in 200 μL olive oil, and orally administered to nude mice for 7 consecutive days after transplantation. Mice were then sacrificed by an overdose of isoflurane. All experimental protocols were approved by the Animal Usage Committee of Keio University (Approval number, 15029-(0)). The methods were carried out in accordance with the approved guidelines.

### Statistical analysis

Data are expressed as average ± SD for in vitro and ± SEM for in vivo experiments. Statistical analyses were conducted using SPSS Statistics software (Version 23 for Macintosh, IBM Inc). A one- or two-way analysis of variance (ANOVA) followed by Tukey’s test was used to evaluate differences between more than three groups. Differences were considered to be significant for values of *P*<0.05.

## Results

### Crizotinib specifically induced apoptosis in Ba/F3 cells transformed by NPM-ALK

**Fig 1:**
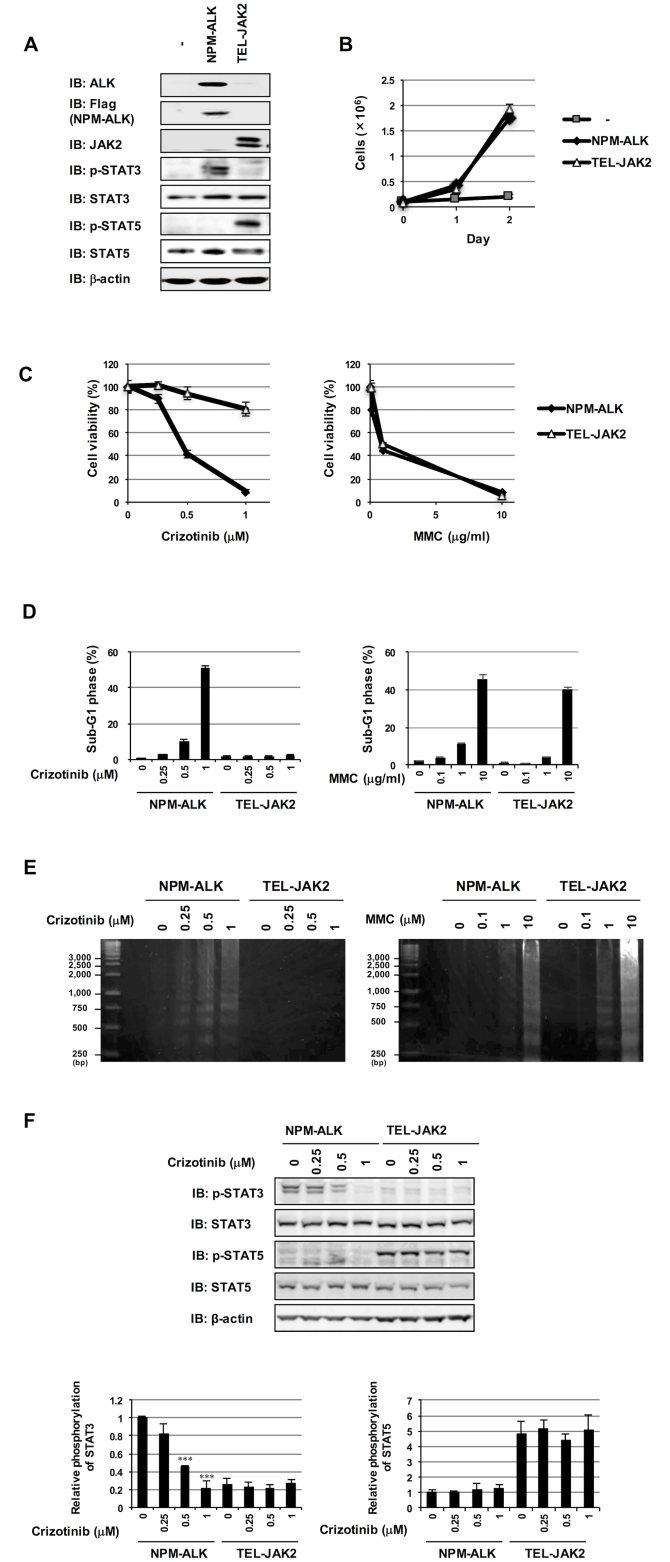
Crizotinib-induced apoptosis in Ba/F3 cells transformed by NPM-ALK. Ba/F3 cells were infected with an empty virus (-) and retrovirus expressing NPM-ALK or TEL-JAK2. (A) Whole cell lysates were immunoblotted with an anti-ALK antibody, anti-Flag antibody, anti-JAK2 antibody, anti-phospho-STAT3 antibody, anti-STAT3 antibody, anti-phospho-STAT5 antibody, anti-STAT5 antibody or anti-β-actin antibody. (B) Transduced Ba/F3 cells were incubated with RPMI containing 10% FBS in the absence of IL-3 for 2 days. Viable cell numbers at 24 hr and 48 hr were counted by the Trypan blue exclusion method. Values are the mean ± SD of three independent experiments. (C-F) Transduced Ba/F3 cells were incubated with RPMI containing 10% FBS in the presence of crizotinib (0.25, 0.5, 1 μM) and MMC (0.1, 1, 10 μg/mL) for 24 hr. (C) Cell viability was measured by the WST assay. Values are the mean ± S.D. of four independent experiments. (D) Cells were fixed, treated with propidium iodide, and subjected to a flow cytometric analysis. The percentages of cells in the sub-G1 phase were graphed. Values are the mean ± S.D. of four independent experiments. (E) To evaluate the apoptotic cell death, the chromatin DNA was isolated from cells and subjected to agarose gel electrophoresis. (F) Whole cell lysates were prepared and the phosphorylation of STAT3 and STAT5 was analyzed by immunoblotting. The relative phosphorylation level of STAT3 and STAT5 is shown in the graphs. Values are given as the mean ± SD of three independent experiments. ***P < 0.001 significantly different from control group of Ba/F3 cells expressing NPM-ALK.

Similar to NPM-ALK, the oncogenic tyrosine kinase, TEL-JAK2 is generated from reciprocal chromosomal translocations and causes lymphoma and leukemia [17–19]. In order to examine the specificity of the drug efficacy of crizotinib against tumor-related tyrosine kinases, murine hematopoietic Ba/F3 cells were infected with an empty retrovirus (-) and retroviruses harboring NPM-ALK and TEL-JAK2, respectively. NPM-ALK induced the phosphorylation of STAT3, whereas TEL-JAK2 induced the phosphorylation of STAT5 (Fig 1A). NPM-ALK and TEL-JAK2 both induced the cytokine-independent proliferation of Ba/F3 cells (Fig 1B). We then investigated the effects of the ALK inhibitor, crizotinib on the viability of Ba/F3 cells transformed by NPM-ALK and TEL-JAK2. As shown in Fig 1C, crizotinib specifically decreased the viability of Ba/F3 cells expressing NPM-ALK in a dose-dependent manner, while Ba/F3 cells expressing TEL-JAK2 appeared to exhibit resistance against the treatment with crizotinib. On the other hand, mitomycin C (MMC), a DNA crosslinking anti-cancer drug, significantly decreased the viabilities of Ba/F3 cells expressing NPM-ALK and TEL-JAK2 (Fig 1C, right). The treatment of Ba/F3 cells expressing NPM-ALK with crizotinib resulted in the accumulation of cells in the sub-G1 phase, which is well established as a characteristic of apoptotic cell death; however, this was not observed in cells expressing TEL-JAK2. In contrast, MMC significantly increased the percentages of both cells in the sub-G1 phase (Fig 1D). To confirm that crizotinib induces the apoptotic cell death, we investigated whether internucleosomal DNA fragmentation, a biochemical characteristic of apoptosis, could be detected in the crizotinib-treated cells. As shown in Fig 1E, the treatment with crizotinib clearly induced the ladder pattern of DNA internucleosomal fragmentation in Ba/F3 cells expressing NPM-ALK but not Ba/F3 cells expressing TEL-JAK2. On the other hand, MMC induced DNA internucleosomal fragmentation in both cells (Fig 1E). The treatment with crizotinib inhibited STAT3 phosphorylation in a dose-dependent manner in Ba/F3 cells expressing NPM-ALK, but had no effect on STAT5 phosphorylation in Ba/F3 cells expressing TEL-JAK2 (Fig 1F). These results suggest that crizotinib specifically induced apoptosis in cells transformed by NPM-ALK.

### α-Tocopherol rescued crizotinib-induced apoptosis in cells transformed by NPM-ALK in a ROS-independent manner

**Fig 2:**
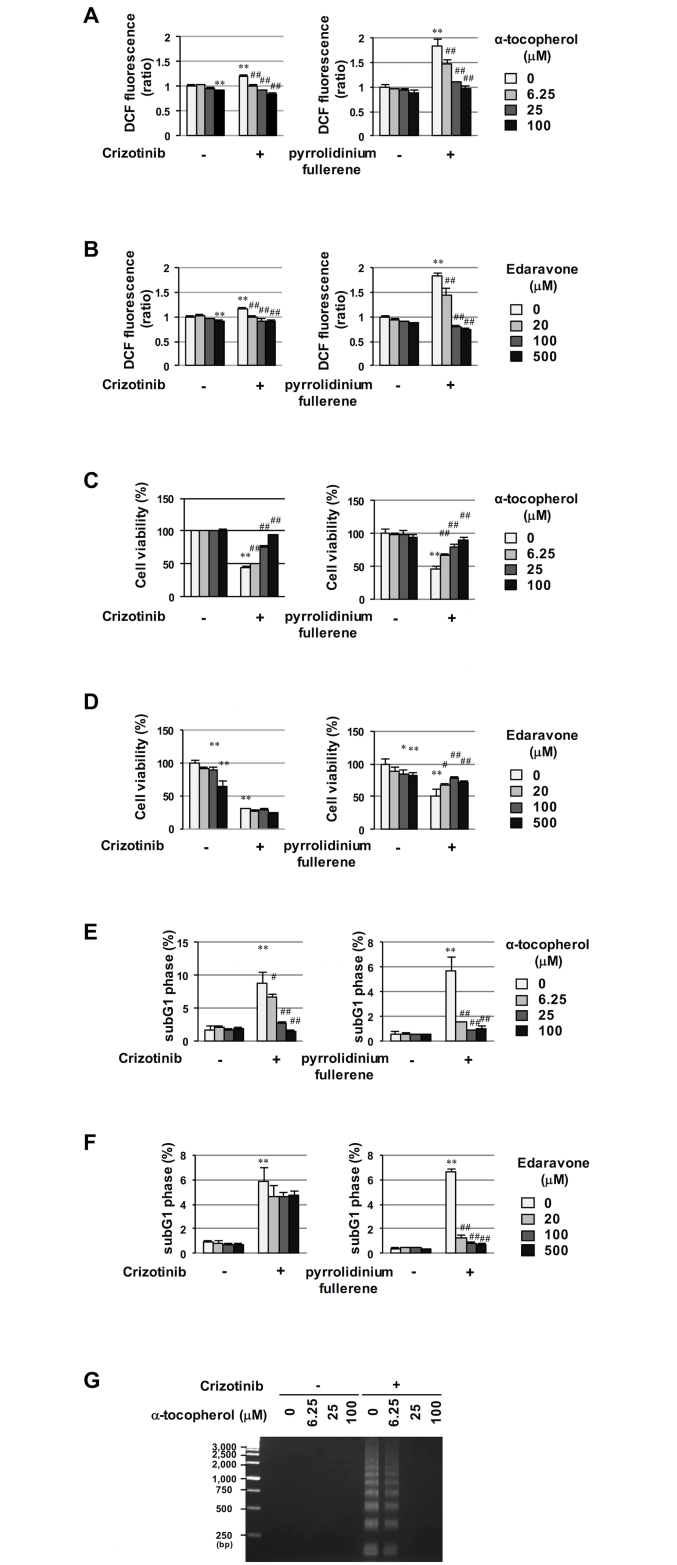
Attenuation of effects of crizotinib by α-tocopherol in a ROS-independent manner. (A-F) Ba/F3 cells expressing NPM-ALK were treated with crizotinib (0.5 μM) or pyrrolidinium fullerene (25 μM) in combination with α-tocopherol (6.25, 25, and 100 μM) or edaravone (20, 100, and 500 μM) for 24 hr. (A, B) Intracellular ROS levels were measured in a flow cytometric analysis using DCFH-DA. (C, D) Cell viabilities were measured by a WST assay. (E, F) The cell cycle, also including the accumulation of sub-G1 phase cells, was examined in a flow cytometric analysis. (A-F) Values are given as the mean ± SD of three independent experiments. **P < 0.01; *P < 0.05 significantly different from the control group; ^##^*P* < 0.01; ^#^*P* < 0.05 significantly different from the group incubated with 0.5 μM crizotinib or 25 μM pyrrolidinium fullerene. (G) To evaluate the apoptotic cell death, the chromatin DNA was isolated from cells and subjected to agarose gel electrophoresis.

A previous study reported that crizotinib increases intracellular ROS levels in human alveolar rhabdomyosarcoma and embryonal rhabdomyosarcoma cells [13]. Therefore, we evaluated the generation of intracellular ROS by measuring oxidized DCFH-DA (2′,7′-dichlorodihydrofluorescein-DA), and tested whether crizotinib induces apoptosis in Ba/F3 cells expressing NPM-ALK through the generation of ROS. In a previous study, pyrrolidinium fullerene caused apoptotic cell death via the generation of ROS in HL60 cells [14]; therefore, we utilized it as a positive control for ROS generation. As shown in Fig 2A, while the treatment with pyrrolidinium fullerene drastically enhanced the generation of ROS in Ba/F3 cells expressing NPM-ALK, the treatment with crizotinib slightly enhanced the generation of ROS. In addition, the co-treatment with two kinds of anti-oxidants, α-tocopherol and edaravone significantly suppressed pyrrolidinium fullerene and crizotinib-induced ROS generation in a dose-dependent manner (Fig 2A and 2B). We then investigated the effects of these anti-oxidants on the viability of Ba/F3 cells expressing NPM-ALK, which were treated with crizotinib or pyrrolidinium fullerene. Similar to the results shown in Fig 1, crizotinib markedly reduced the viability of Ba/F3 cells expressing NPM-ALK; however, α-tocopherol, but not edaravone significantly recovered the viability of crizotinib-treated cells (Fig 2C and 2D). On the other hand, the pyrrolidinium fullerene-induced reduction in cell viability appeared to be canceled by the co-treatment with α-tocopherol and edaravone (Fig 2C and 2D). These results clearly suggest that crizotinib-induced cell death and its cancelation by α-tocopherol were the most unlikely to depend on intracellular ROS levels. We subsequently examined the effects of anti-oxidants on the crizotinib and pyrrolidinium fullerene-induced accumulation of sub-G1 phase cells in Ba/F3 cells expressing NPM-ALK. As shown in Fig 2E and 2F, α-tocopherol, but not edaravone consistently reduced increases in the accumulation of sub-G1 phase in Ba/F3 cells expressing NPM-ALK induced by crizotinib. On the other hand, the pyrrolidinium fullerene-induced accumulation of sub-G1 phase cells was reduced by the treatment with α-tocopherol and edaravone. In addition, the crizotinib-induced internucleosomal DNA fragmentation was prevented by the co-treatment with α-tocopherol (Fig 2G). Collectively, these results strongly suggest that ROS are not critical for crizotinib-induced apoptosis in cells expressing NPM-ALK. They also indicate that α-tocopherol rescued crizotinib-induced apoptosis in cells transformed by NPM-ALK regardless of its ability to scavenge ROS.

### α-Tocopherol specifically rescued crizotinib-induced apoptotic cell death in cells transformed by NPM-ALK

**Fig 3:**
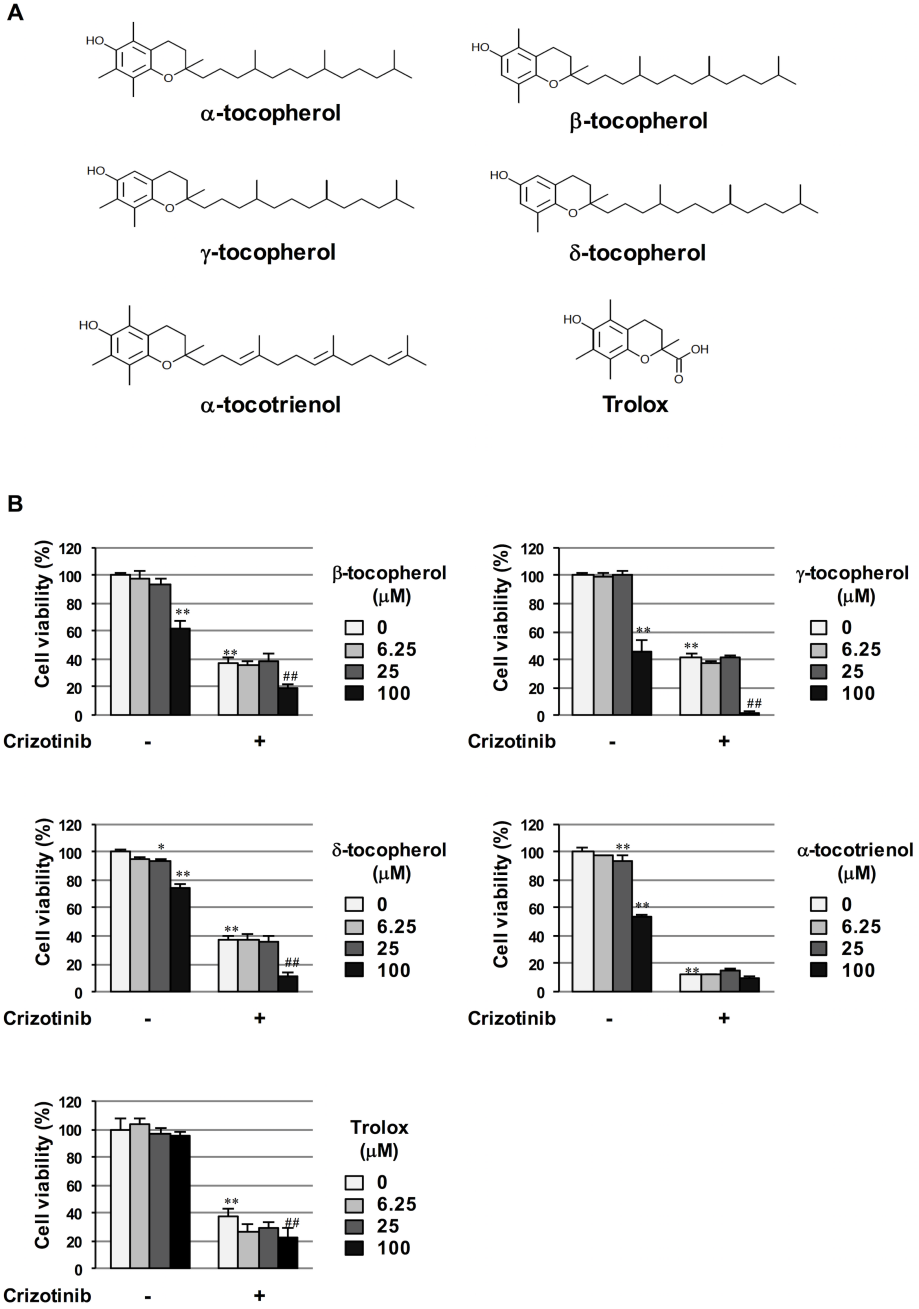
Specific inhibitory effects of α-tocopherol on crizotinib-induced cytotoxicity against Ba/F3 cells expressing NPM-ALK. (A) The chemical structures of α-tocopherol, β-tocopherol, γ-tocopherol, δ-tocopherol, α-tocotrienol, and Trolox are shown. (B) Ba/F3 cells expressing NPM-ALK were treated with crizotinib (0.5 μM) or in combination with β-tocopherol, γ-tocopherol, δ-tocopherol, α-tocotrienol or Trolox (6.25, 25, and 100 μM) for 24 hr. Cell viabilities were measured by a WST assay. Values are given as the mean ± SD of four independent experiments. **P < 0.01; *P < 0.05 significantly different from the control group; ^##^*P* < 0.01 significantly different from the group incubated with 0.5 μM crizotinib.

Vitamin E is an antioxidant that includes two groups of fat-soluble benzopyranol compounds: tocopherols and tocotrienols [20]. Based on their chemical structures, tocopherols are a series of related benzopyranols with a C16-saturated side chain, while tocotrienols contain three double bonds on the C16 side chain. Each group contains four constituents termed α (5,7,8-trimethyl), β (5,8-dimethyl), γ (7,8-dimethyl), and δ (8-methyl) (Fig 3A). We investigated whether other vitamin E-related compounds such as other tocopherols, α-tocotrienol, and trolox exert inhibitory effects on crizotinib-induced cell death in cells transformed by NPM-ALK similar to α-tocopherol. However, β-tocopherol, γ-tocopherol, δ-tocopherol, α-tocotrienol, and trolox failed to inhibit crizotinib-induced cytotoxicity in Ba/F3 cells expressing NPM-ALK (Fig 3B). These results clearly demonstrate that α-tocopherol harbors a unique characteristic as not only an anti-oxidant, but also a specific chemical suppressor of crizotinib-induced cytotoxicity in NPM-ALK-positive tumor cells.

### α-Tocopherol indirectly attenuated the inhibition of NPM-ALK phosphorylation by crizotinib

**Fig 4:**
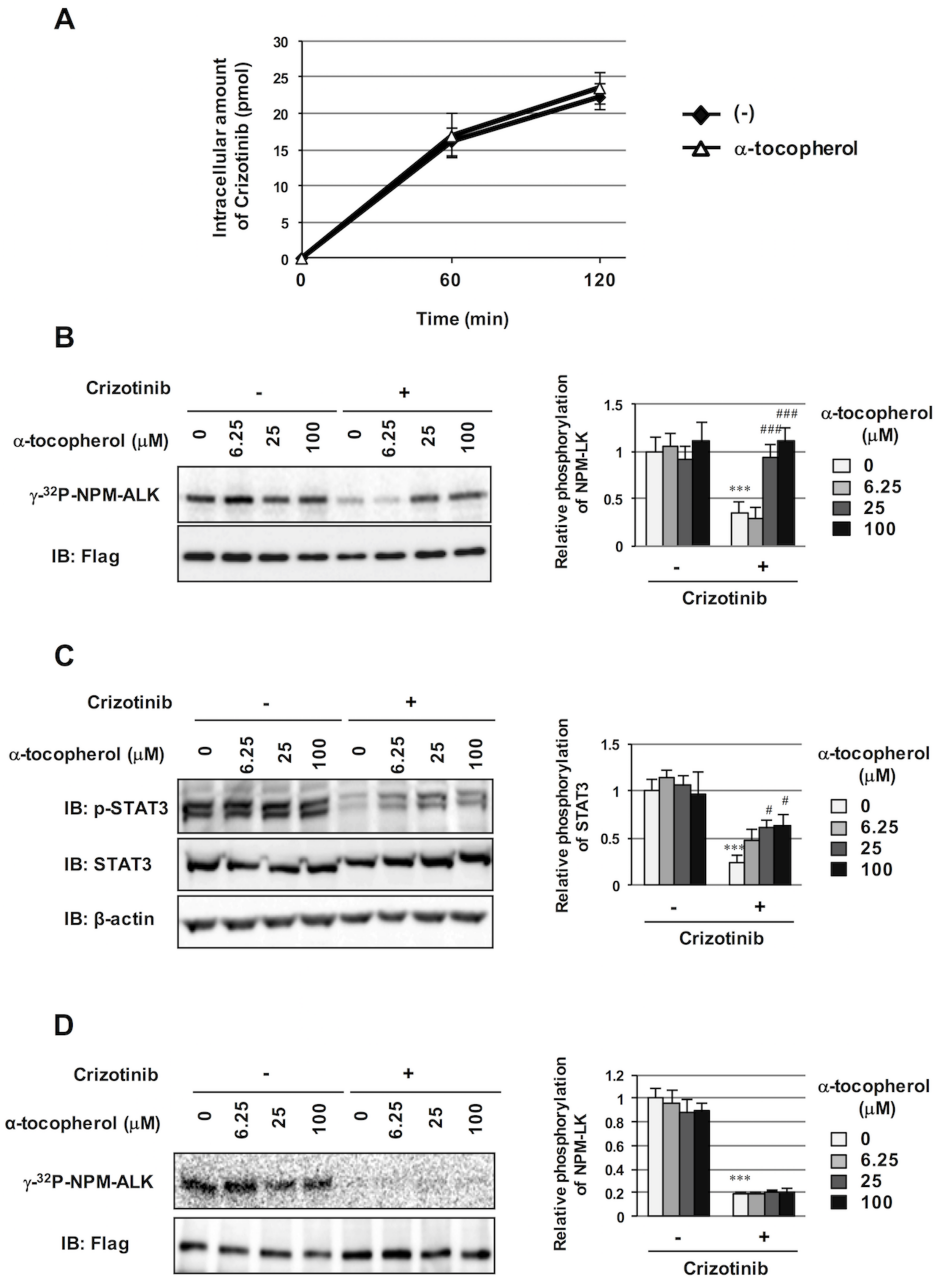
Indirect attenuation of the crizotinib-induced inhibition of ALK activity by α-tocopherol. (A) Ba/F3 cells expressing NPM-ALK were incubated with crizotinib (0.5 μM) in combination with α-tocopherol (25 μM) for 60 min and 120 min. The concentration of intracellular crizotinib was measured by LC/MS. (B, C) Ba/F3 cells expressing NPM-ALK were incubated with crizotinib (0.5 μM) in combination with α-tocopherol (6.25, 25, 100 μM) for 24 hr. Values are given as the mean ± SD of three independent experiments. (B) NPM-ALK were immunoprecipitated with an anti-Flag antibody from cell lysates. NPM-ALK immunoprecipitates were assayed for kinase activity and phosphorylated NPM-ALK was detected by autoradiography (upper). The immunoprecipitates were immunoblotted with anti-Flag antibody (bottom). The relative phosphorylation level of NPM-ALK is shown in the graph. Values are given as the mean ± SD of three independent experiments. ***P < 0.001 significantly different from the control group; ^###^*P* < 0.001 significantly different from the group incubated with 0.5 μM crizotinib. (C) Cell lysates were immunoblotted with an anti-phospho-STAT3 antibody (Tyr705), anti-STAT3 antibody, or anti-β-actin antibody. The relative phosphorylation level of STAT3 is shown in the graph. Values are given as the mean ± SD of three independent experiments. ***P < 0.001 significantly different from the control group; ^#^*P* < 0.05 significantly different from the group incubated with 0.5 μM crizotinib. (D) Ba/F3 cells expressing NPM-ALK were lysed and the NPM-ALK protein was immunoprecipitated with an anti-Flag antibody. Immunoprecipitated NPM-ALK was incubated with crizotinib (0.5 μM) in combination with α-tocopherol (6.25, 25, 100 μM) for 15 min, and then assayed for kinase activity. Phosphorylated NPM-ALK was detected by autoradiography (upper). The immunoprecipitates were immunoblotted with anti-Flag antibody (bottom). The relative phosphorylation level of NPM-ALK is shown in the graph. Values are given as the mean ± SD of three independent experiments. ***P < 0.001 significantly different from the control group.

In order to elucidate the mechanisms by which α-tocopherol specifically attenuates the effects of crizotinib, we examined whether the uptake of crizotinib into Ba/F3 cells expressing NPM-ALK was affected by α-tocopherol. After the treatment with crizotinib in the absence and presence of α-tocopherol, the intracellular concentration of crizotinib was measured by LC-MS. However, similar amounts of crizotinib were taken up by cells regardless of the co-treatment with α-tocopherol (Fig 4A). We then investigated whether α-tocopherol attenuated crizotinib-induced inhibitory effects on kinase activity by NPM-ALK. Cells expressing NPM-ALK were treated with crizotinib in the presence or absence of α-tocopherol, and NPM-ALK was then immunoprecipitated with the anti-Flag antibody in order to evaluate its kinase activity using an *in vitro* kinase assay with radioactive ATP. The autophosphorylation of NPM-ALK was completely suppressed by crizotinib, and this crizotinib-induced inhibition was canceled by the co-treatment with α-tocopherol in a dose-dependent manner (Fig 4B). Furthermore, the treatment with α-tocopherol markedly attenuated the crizotinib-induced inhibition of STAT3 phosphorylation (Fig 4C). In an attempt to gain further insights into the mechanisms by which α-tocopherol suppressed crizotinib-induced inhibitory effects on NPM-ALK, we tested whether α-tocopherol canceled the inhibition of NPM-ALK by crizotinib *in vitro*. Immunoprecipitated NPM-ALK was treated with crizotinib in the presence or absence of α-tocopherol. As shown in Fig 4D, although crizotinib completely inhibited the phosphorylation of immunoprecipitated NPM-ALK, the co-treatment with α-tocopherol failed to attenuate the inhibitory effects of crizotinib. These results suggest that the cancelation of the effects of crizotinib by α-tocopherol was the most unlikely to be due to the direct effects of α-tocopherol on the NPM-ALK protein.

### The co-treatment with α-tocopherol swept the effect of crizotinib on the subcellular localization of NPM-ALK

**Fig 5:**
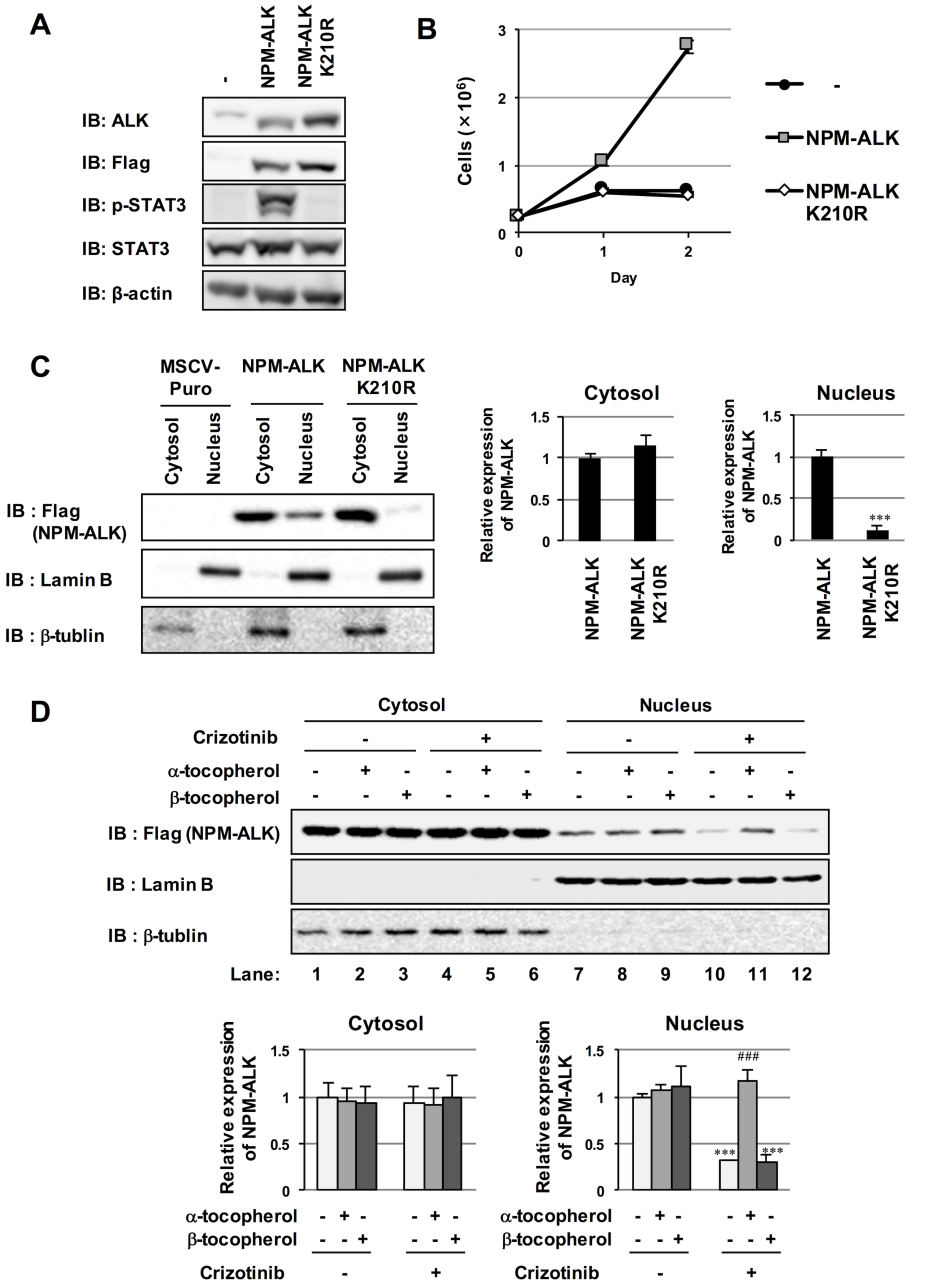
The effects of treatment with crizotinib and co-treatment with crizotinib and α-tocopherol on the nuclear localization of NPM-ALK. (A-C) Ba/F3 cells were infected with an empty vector (MSCV-puro) and retroviruses encoding N-Flag NPM-ALK and its kinase dead mutant (K210R). (A) Transduced Ba/F3 cells were incubated without IL-3 for 24 hr. Whole cell lysates were immunoblotted with an anti-ALK antibody, anti-Flag antibody, anti-phspho-STAT3 (Y705) antibody, anti-STAT3 antibody, or anti-β-actin antibody. (B) Transduced Ba/F3 cells were incubated without IL-3 and counted each day for 2 days by Trypan blue staining. Values are given as the mean ± SD of three independent experiments. (C) Cytosol fraction and nuclear fraction were prepared and immunoblotted with anti-Flag antibody, anti-Lamin B or anti-β-tublin. The relative expression levels of NPM-ALK and NPM-ALK (K210R) in cytosol and nucleus are shown in the graphs. Values are given as the mean ± SD of three independent experiments. ***P < 0.01 significantly different from the group of NPM-ALK. (D) Ba/F3 cells expressing NPM-ALK were treated with crizotinib (0.5 μM) or in combination with α-tocopherol (25 μM) or β-tocopherol (25 μM) for 24 hr. Cytosol fraction and nuclear fraction were prepared and immunoblotted with anti-Flag antibody, anti-Lamin B or anti-β-tublin. The relative expression levels of NPM-ALK in cytosol and nucleus are shown in the graphs. Values are given as the mean ± SD of three independent experiments. ***P < 0.01 significantly different from the control group; ^###^*P* < 0.001 significantly different from the group incubated with 0.5 μM crizotinib.

Ba/F3 cells were infected with an empty retrovirus (-) and retroviruses harboring NPM-ALK and its kinase dead mutant, in which the lysine residue at 210 in the ATP-binding site was mutated to arginine (K210R). As shown in Fig 5A and 5B, while NPM-ALK induced the phosphorylation of STAT3 and cytokine-independent proliferation, NPM-ALK (K210R) failed to induce STAT3 activation and confer cytokine independence to Ba/F3 cells. It has been reported that NPM-ALK was localized both in cytosol and nucleus [5,21–23]. We investigated the subcellular localization of NPM-ALK and NPM-ALK (K210R). Interestingly, whereas NPM-ALK was localized in both cytosol and nucleus in Ba/F3 cells, the nuclear localization of NPM-ALK (K210R) was completely disappeared (Fig 5C), suggesting that kinase activity of NPM-ALK affected its cellular localization. Strikingly, the nuclear localization of NPM-ALK was clearly disappeared by the treatment with crizotinib (Fig 5D, comparing lane 7 and lane 10), suggesting the correlation between the subcellular localization of NPM-ALK and its kinase activity. Next, we investigated whether the co-treatment with α-tocopherol affects the effect by crizotinib. Interestingly, the co-treatment with α-tocopherol swept the effect of crizotinib on the subcellular localization of NPM-ALK (Fig 5D, comparing lane 10 and lane 11). On the other hand, β-tocopherol failed to exhibit the cancelation effect against crizotinib (Fig 5D, comparing lane 10 and lane 12).

### α-Tocopherol significantly attenuated the anti-tumor activity of crizotinib in nude mice transplanted with cells expressing NPM-ALK

**Fig 6:**
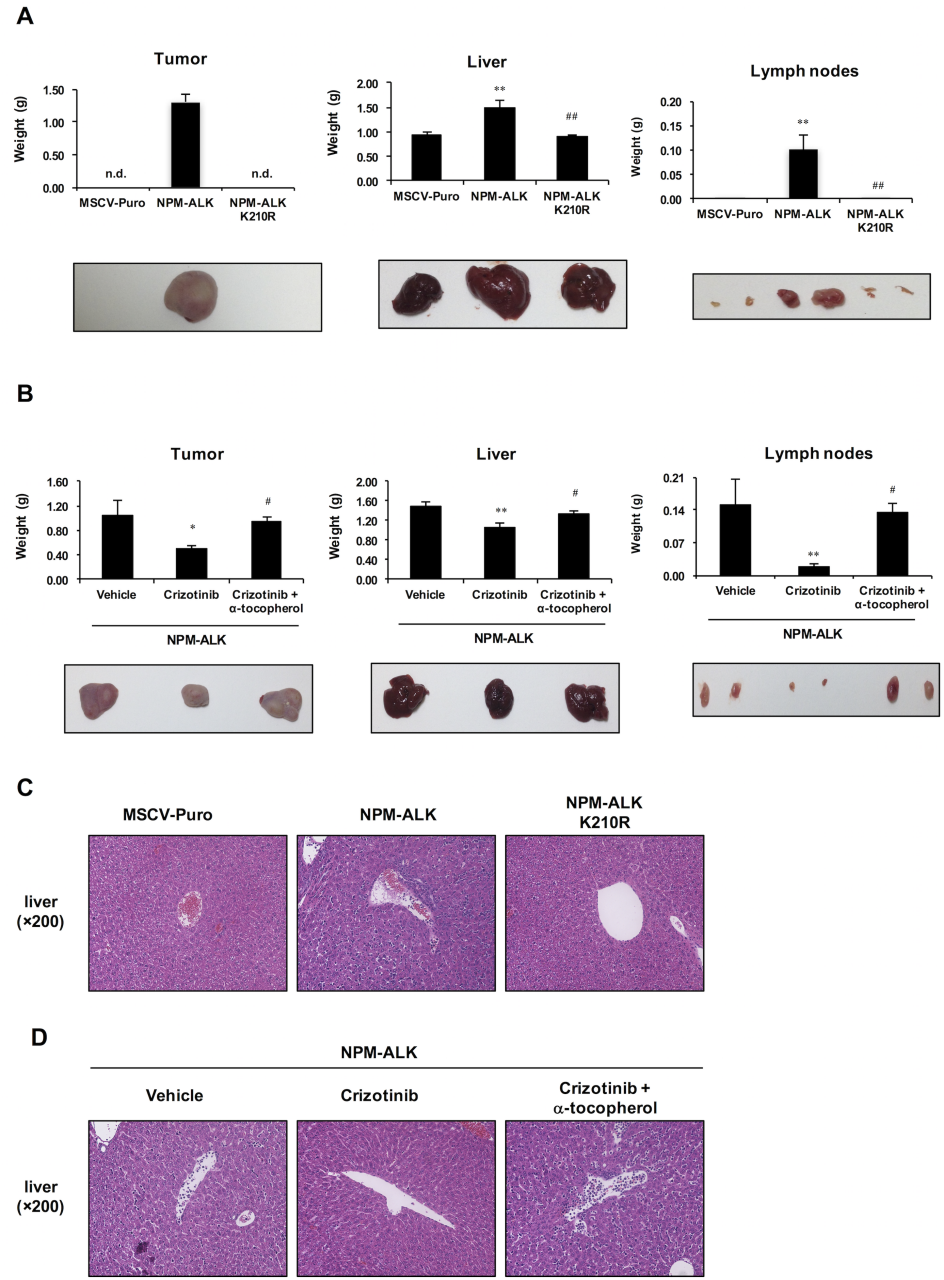
Attenuation of the anti-tumor activity of crizotinib by α-tocopherol in nude mice inoculated with Ba/F3 cells expressing NPM-ALK. (A) Ba/F3 cells were infected with an empty virus (MSCV-Puro) and retroviruses expressing NPM-ALK and its kinase dead mutant (K210R). Transduced Ba/F3 cells (2×10^7^ cells) were s.c. injected into nude mice. The weights of tumor, liver, and lymph node were measured 16 days post-inoculation. Values are given as the mean ± SEM of five independent experiments. **P < 0.01 significantly different from the group of mice inoculated with empty virus-infected cells; ^##^*P* < 0.01 significantly different from the group of mice inoculated with cells expressing NPM-ALK; n.d., not detected. (B) Nude mice were transplanted with Ba/F3 cells expressing NPM-ALK and orally administered crizotinib (1 mg/mouse) in combination with/without α-tocopherol (4 mg/mouse) for 7 consecutive days. Tumor, liver, and lymph node weights were measured 16 days post-inoculation. Values are given as the mean ± SEM of three independent experiments. **P < 0.01; *P < 0.05 significantly different from the vehicle-treated group; ^#^*P* < 0.05 significantly different from the crizotinib-treated group. (C, D) Sixteen days post-inoculation, liver sections were stained with hematoxylin and eosin (magnification: ×400).

In order to evaluate the anti-tumor effects of crizotinib and its cancellation by α-tocopherol, we attempted to establish an assay system by subcutaneously (s.c.) inoculating transduced Ba/F3 cells expressing NPM-ALK into nude mice. As shown in Fig 6A, whereas Ba/F3 cells expressing NPM-ALK exhibited significant tumor-forming activity, tumor formation was not observed in nude mice inoculated with Ba/F3 cells expressing NPM-ALK (K210R). In these nude mice, abnormally enlarged livers and lymph nodes were observed when mice were transplanted with Ba/F3 cells expressing NPM-ALK, but not NPM-ALK (K210R), suggesting that the kinase activity of NPM-ALK is required for tumorigenesis (Fig 6A). We then tested the *in vivo* effects of crizotinib and α-tocopherol on NPM-ALK-induced tumorigenesis. Nude mice were orally administered crizotinib (1 mg/mouse) and α-tocopherol (4 mg/mouse) for 7 consecutive days after the transplantation of Ba/F3 cells expressing NPM-ALK. The dosages of crizotinib and α-tocopherol for the treatment of mice were established by correlative calculations according to the effective dose in human patients with NSCLC and tolerable upper intake levels (ULs) for adults aged 19 years and older [24–26]. In contrast to mice treated with vehicle, tumor formation and enlargements in the liver and lymph nodes were effectively suppressed by the administration of crizotinib. On the other hand, the simultaneous administration of crizotinib and α-tocopherol significantly attenuated the inhibitory effects of crizotinib on tumor formation and enlargements in the liver and lymph nodes in mice receiving Ba/F3 cells expressing NPM-ALK (Fig 6B). In addition, liver sections were prepared and stained with hematoxylin and eosin. The density of cells was significantly greater, and the arrangement of hepatocytes was disrupted more in the livers of mice inoculated with Ba/F3 cells expressing NPM-ALK than in the livers of mice inoculated with control Ba/F3 cells and Ba/F3 cells expressing the kinase dead mutant of NPM-ALK (K210R) (Fig 6C), suggesting that these differences were due to the infiltration of NPM-ALK-induced tumor cells. We then attempted to investigate the effects of crizotinib and α-tocopherol on these abnormalities in the livers of mice transplanted with Ba/F3 expressing NPM-ALK. The density of cells and disarrangement of hepatocytes were significantly less with the treatment with crizotinib than with the vehicle. However, in the livers of mice simultaneously administered α-tocopherol, abnormalities such as the enhanced density and disarrangement of hepatocytes were similar to those in the livers of mice treated with vehicle (Fig 6D). Taken together, our results strongly support crizotinib being useful for the treatment of NPM-ALK-related tumors; however, its therapeutic effects will be abrogated by a co-treatment with α-tocopherol.

### α-Tocopherol significantly attenuated the effect of crizotinib on NPM-ALK-positive cells derived from human ALCL patients

**Fig 7:**
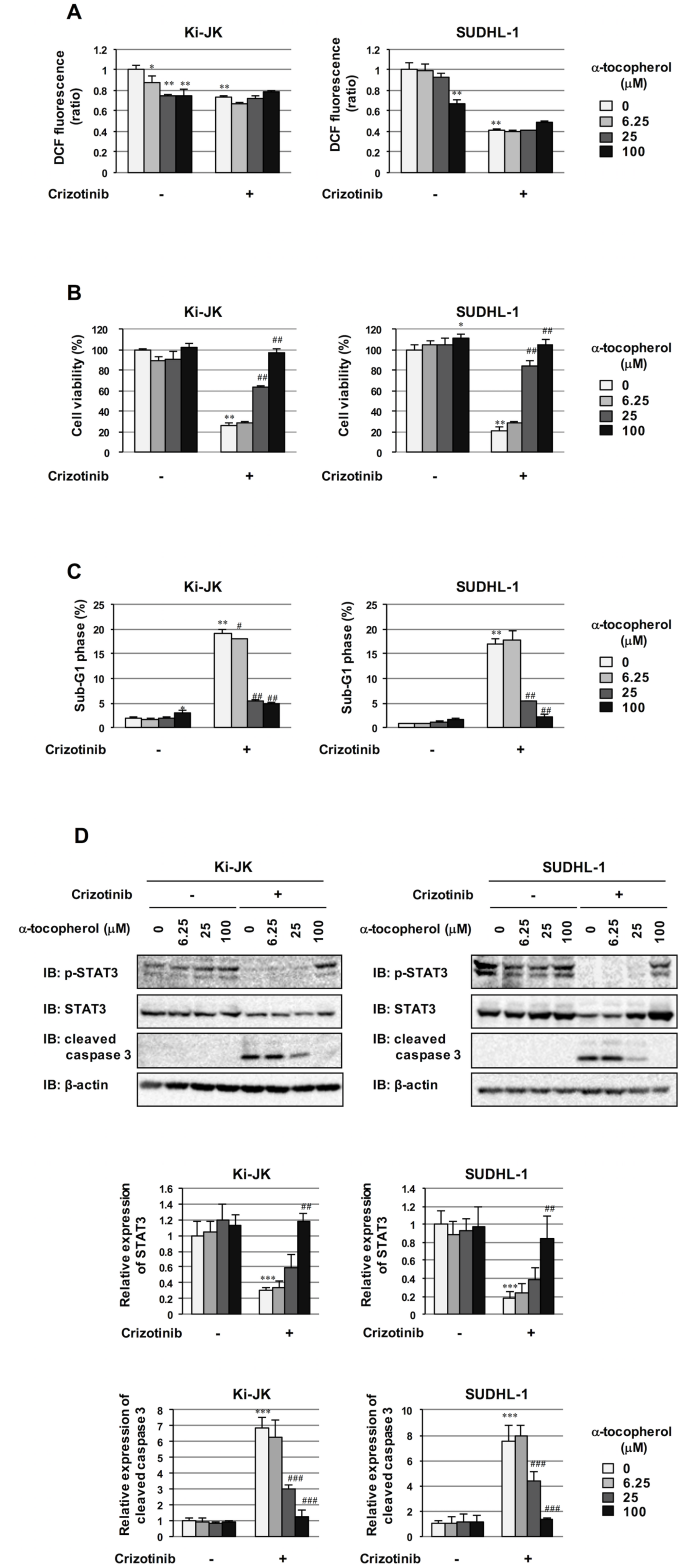
Attenuation of effects of crizotinib by α-tocopherol in NPM-ALK-positive cells derived from human ALCL patients. Ki-JK and SUDHL-1 cells were treated with crizotinib (0.5 μM) in combination with α-tocopherol (6.26, 25, and 100 μM) for 24 hr. (A) Intracellular ROS levels were measured in a flow cytometric analysis using DCFH-DA. (B) Cell viabilities were measured by a WST assay. (C) The cell cycle, also including the accumulation of sub-G1 phase cells, was assessed in a flow cytometric analysis. (A-C) Analytical values are given as the mean ± SD of three independent experiments. **P < 0.01; *P < 0.05 significantly different from the control group; ^##^*P* < 0.01; ^#^*P* < 0.05 significantly different from the group incubated with 0.5 μM crizotinib. (D) Cell lysates were immunoblotted with an anti-phospho-STAT3 antibody (Tyr705), anti-STAT3 antibody, anti-cleaved caspase 3 antibody or anti-β-actin antibody. The relative phosphorylation level of STAT3 and the relative expression level of cleaved caspase 3 are shown in the graphs. Values are given as the mean ± SD of three independent experiments. ***P < 0.001 significantly different from the control group; ^###^*P* < 0.001; #^#^*P* < 0.01 significantly different from the group incubated with 0.5 μM crizotinib.

Ki-JK and SUDHL-1 cell lines were derived from ALCL patients [27, 28], which have a t(2;5)(p23;q35) translocation producing NPM-ALK. We examined whether α-tocopherol suppresses the inhibitory effects of crizotinib on the proliferation of Ki-JK cells and SUDHL-1 cells. These cell lines were treated with crizotinib in the presence and absence of α-tocopherol, and intracellular ROS levels, cell viability, and the percentages of cells in the sub-G1 phase were then evaluated. As shown in Fig 7A, the amounts of intracellular ROS were slightly reduced when these two cell lines were treated with a high dose of α-tocopherol. On the other hand, the treatment with crizotinib reduced intracellular ROS levels slightly in Ki-JK cells and markedly in SUDHL-1 cells; however, α-tocopherol did not have any effects on crizotinib-induced reductions in ROS levels in these cells (Fig 7A). Although crizotinib markedly decreased the viabilities of Ki-JK cells and SUDHL-1 cells, α-tocopherol significantly rescued these cells from crizotinib-induced cytotoxicity in a dose-dependent manner (Fig 7B). In addition, crizotinib significantly induced the accumulation of sub-G1 phase cells in these cell lines; however, this effect was canceled by the treatment with α-tocopherol (Fig 7C). Although crizotinib inhibited the phosphorylation of STAT3, the treatment with α-tocopherol markedly attenuated the crizotinib-induced inhibition of STAT3 phosphorylation in both Ki-JK cells and SUDHL-1 cells. Furthermore, crizotinib induced the activation of caspase 3, the treatment with α-tocopherol markedly attenuated the crizotinib-induced activation of caspase 3 in both Ki-JK cells and SUDHL-1 cells (Fig 7D), suggesting that crizotinib-induced apoptosis was canceled by the treatment with α-tocopherol. These results demonstrate that the anti-tumor activity of crizotinib against NPM-ALK is also canceled by α-tocopherol in cells derived from human patients with ALCL.

## Discussion

The results of the present study strongly support the usefulness of crizotinib in the treatment of NPM-ALK-related tumors. Crizotinib is an orally available ALK inhibitor that inhibits ALK by competitively binding within the ATP-binding pocket, and also inhibits receptor tyrosine kinases including c-Met and ROS1 [9, 10]. We demonstrated that the simultaneous administration of α-tocopherol markedly inhibited the therapeutic efficacy of crizotinib; however, we were unable to elucidate the precise mechanism by which α-tocopherol abrogates the inhibitory effects of crizotinib on NPM-ALK.

In the present study, we demonstrated that other anti-oxidants including edaravone, β-tocopherol, δ-tocopherol, γ-tocopherol, α-tocotrienol, and Trolox failed to cancel the effects of crizotinib (Figs 1 and 3), suggesting that α-tocopherol inhibits the anti-tumor effects of crizotinib in a ROS level-independent manner. It has been shown that α-tocopherol negatively regulates the proliferation of vascular smooth muscle cells and aggregation of platelets by inhibiting PKC independently of its antioxidant properties [29]. Previous findings demonstrated that the α-tocopherol-induced inhibition of PKC was suppressed by a co-treatment with β-tocopherol when administered at a similar concentration to that of α-tocopherol [30]. We examined the effects of a co-treatment with α-tocopherol and β-tocopherol on cytotoxicity induced by crizotinib in Ba/F3 cells expressing NPM-ALK. The results obtained revealed that β-tocopherol failed to buffer the inhibitory effects of α-tocopherol on crizotinib-induced cytotoxicity (S1 Fig). A previous study reported that α-tocopherol specifically binds to the α-tocopherol transfer protein (TTP) and is involved in the intracellular trafficking of α-tocopherol [31]. α-Tocopherol has also been reported to activate p38 MAP kinase and its downstream transcription factor, MITF in order to positively regulate the function of osteoclasts in bone tissue [32]. In an attempt to clarify the functional involvement of p38 and TTP in the effects of α-tocopherol, we utilized the p38 inhibitor, SB203580, and siRNA against TTP. Although the treatment with SB203580 reduced the viability of Ba/F3 cells expressing NPM-ALK, the α-tocopherol-induced cancelation of cytotoxicity induced by crizotinib was not affected by SB203580 or the knockdown of TTP (S2 Fig), suggesting that p38 and TTP are not involved in the inhibitory effects of α-tocopherol on crizotinib-induced cytotoxicity.

Interestingly, higher levels of α-tocopherol were needed for rescuing STAT3 phosphorylation in human ALCL cell lines compared to Ba/F3 cells expressing NPM-ALK (Figs 4C and 7D). Although IC_50_ of crizotinib against c-Met and ROS is higher than against ALK, crizotinib inhibits not only ALK, but also c-Met and ROS [9, 10]. Therefore, it is suggested that other tyrosine kinases which induces phosphorylation of STAT3 could be involved in the oncogenicity of ALCL-derived cell lines. Indeed, it was reported that the phosphorylation of STAT3 was not completely inhibited by the knockdown of NPM-ALK using siRNA in SUDHL-1 cells [33]. Although speculative, the involvement of other tyrosine kinases such as c-Met and ROS might be a reason for the requirement of high dosage of α-tocopherol for rescuing STAT3 phosphorylation in SULH-1 cells and Ki-JK cells. Furthermore, whereas crizotinib slightly induced ROS generation in Ba/F3 cells, intracellular ROS levels was decreased by the treatment with crizotinib in human ALCL cell lines (Figs 2B and 7A). Since it was reported that c-Met harbors the ability to control the ROS generation and contributes in the oncogenicity of ALCL-derived cell lines [34, 35], it is also suggested that the involvement of c-Met could be a reason for the different responses against the treatment of crizotinib in between ALCL-derived cell lines and Ba/F3 cells.

As shown in Fig 5, NPM-ALK was localized in the cytosol and the nucleus as previously reported [5, 21, 22]. Remarkably, the nuclear localization of NPM-ALK required its kinase activity, and crizotinib effectively suppressed the nuclear localization of NPM-ALK. Wild type NPM is mainly localized in nucleoli, however some population shuttled between nucleolus, nucleoplasm, and cytosol [23]. Since NPM forms mono-oligomer, it is not surprising that NPM-ALK could interact with NPM. The localization of NPM-ALK is most likely regulated by fused-NPM region and interacted endogenous NPM, therefore partial population of NPM-ALK was detected in nucleolus. Several groups reported that the nucleolar localization of NPM-ALK was not required for the cellular transformation provoked by NPM-ALK [5, 21]. N-terminal NPM of NPM-ALK could be replaced with the portion of the unrelated translocated promoter region (TPR) protein that activates the TPR-MET fusion kinase by mediating dimerization through its leucine zipper motif [5]. Whereas NPM-ALK was localized in nucleolus, nucleoplasm, and cytoplasm, the artificial fusion protein TPR-ALK was detectable in only cytoplasm [5, 21]. In spite of its different localization, TPR-ALK exhibited the comparable transforming activity with NPM-ALK. However, their reports failed to contradict the possibility that undetectable small population of TPR-ALK could be localized in nucleus, and contribute to the cellular transformation. N-terminal NPM region of NPM-ALK contains nuclear export sequence (NES), however lacks nuclear localization sequence (NLS), suggesting that nuclear localization of NPM-ALK should be triggered by interacting protein of NPM-ALK. Although we do not have any direct evidences supporting the possible contribution of nuclear NPM-ALK in Ba/F3 cells to cellular transformation, it will be important to identify the interacting proteins with nuclear NPM-ALK to elucidate the mechanism how NPM-ALK induces the cellular transformation. In addition, the mutation analysis of NES of NPM-ALK will be the clue for the investigation for the function of nuclear NPM-ALK.

Clinical resistance mutations of EML4-ALK to crizotinib including L1196M, C1156Y, G1269A, L1152R, G1202R, F1174C, I1171T, and S1206Y have been reported in patients with NSCLC [3, 36]. Although we have no direct evidence, the treatment with α-tocopherol may have caused structural alterations in NPM-ALK, similar to the NPM-ALK mutants exhibiting resistance to crizotinib. Second generation ALK inhibitors such as alectinib have recently been developed to overcome acquired resistance to crizotinib [37]. We found that α-tocopherol failed to attenuate cytotoxicity by alectinib against Ba/F3 cells expressing NPM-ALK (S3 Fig). In addition, although we investigated the effects of α-tocopherol on the JAK2 inhibitor, ruxolitinib-induced apoptosis in Ba/F3 cells transformed by a constitutive active mutant of JAK2 (V617F), α-tocopherol failed to rescue ruxolitinib-induced apoptosis in these cells (S3 Fig). Therefore, these results suggest that the inhibitory effects of α-tocopherol on crizotinib depend on its chemical structure.

However, it remains unclear whether the α-tocopherol-mediated inactivation of crizotinib occurs enzymatically or is mediated by non-enzymatic steps.

Vitamin E, which is composed of tocopherols and tocotrienols, is one of the most popular supplements in the world; more than 10% of adults take at least 400 IU of vitamin E daily [38]. α-Tocopherol is the major component of vitamin E and is preferentially absorbed by and accumulates in the human body. Although a number of issues have yet to be clarified, our results indicate that the anti-tumor activities of well-known compounds may be abrogated by environmental factors, such as the intake of supplements, in patients with tumor-related diseases.

## Supporting information

S1 Fig Effects of α-tocopherol and/or β-tocopherol on the viability of Ba/F3 cells expressing NPM-ALK treated with crizotinib. Ba/F3 cells expressing NPM-ALK were treated with crizotinib in combination with α-tocopherol (25 μM) and/or β-tocopherol (25 μM) for 24 hr. Cell viabilities were evaluated by a WST assay. Values are given as the mean ± SD of four independent experiments. **P < 0.01
(DOCX)

S2 Fig Effects of SB203580 and the knockdown of TTP on the viability of Ba/F3 cells expressing NPM-ALK treated with crizotinib. (A) Ba/F3 cells expressing NPM-ALK were treated with crizotinib (0.5 μM) in combination with α-tocopherol (25 μM) and/or SB203580 (30 μM) for 24 hr. Cell viabilities were measured by a WST assay. (B, C) Ba/F3 cells expressing NPM-ALK were transfected with control siRNA and siRNA against TTP (si-control, si-TTP). (B) After 48 hr, total RNA was extracted and RT was performed using an oligo (dT)_20_ primer. Quantitative real-time PCR was performed using an iCycler detection system (Bio-Rad, Berkeley, CA, USA). GAPDH mRNA was analyzed as an internal control. Values are the mean ± S.D. of three independent experiments. *P < 0.05 (C) After 48 hr, transfected cells were treated with crizotinib (0.5 μM) in combination with α-tocopherol (6.25, 25, 100 μM) for 24 hr. Cell viabilities were assessed by a WST assay. Values are given as the mean ± SD of four independent experiments. **P < 0.01.
(DOCX)

S3 Fig Effects of α-tocopherol on the viability of Ba/F3 cells expressing NPM-ALK treated with alectinib and the viability of of Ba/F3 cells expressing EpoR and JAK2 V617F mutants treated with ruxolitinib. (A) Ba/F3 cells expressing NPM-ALK were treated with alectinib (0.1 μM) in combination with α-tocopherol (6.25, 25, and 100 μM) for 24 hr. Cell viabilities were evaluated by a WST assay. Values are given as the mean ± SD of four independent experiments. **P < 0.01 (B) Ba/F3 cells expressing the erythropoietin receptor (EpoR) and JAK2 V617F mutant were treated with ruxolitinib (0.3 μM) in combination with α-tocopherol (6.25, 25, 100 μM) for 24 hr. Cell viabilities were evaluated by a WST assay. Values are given as the mean ± SD of four independent experiments. **P < 0.01 significantly different from the control group; ^##^*P* < 0.01 significantly different from the group incubated with 0.3 μM ruxolitinib.
(DOCX)
