# Supplementary figures and images for: Alpha-tocopherol attenuates the anti-tumor activity of crizotinib against cells transformed by NPM-ALK
Source: PLoS One. 2017 Aug 14;12(8):e0183003. doi: 10.1371/journal.pone.0183003 (PMC5555621; doi:10.1371/journal.pone.0183003)

**
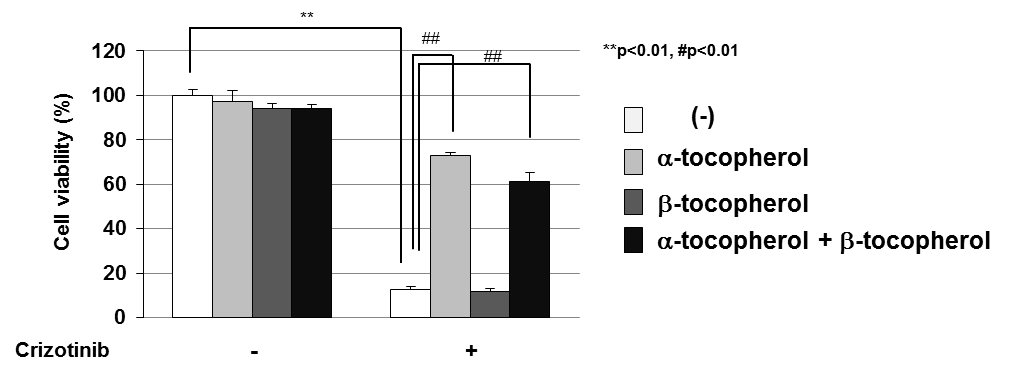
**

Supplement: S1 Fig — Ba/F3 cells expressing NPM-ALK were treated with crizotinib in combination with α-tocopherol (25 μM) and/or β-tocopherol (25 μM) for 24 hr. Cell viabilities were evaluated by a WST assay. Values are given as the mean ± SD of four independent experiments. **P < 0.01 (DOCX) [file pone.0183003.s001.docx]

**
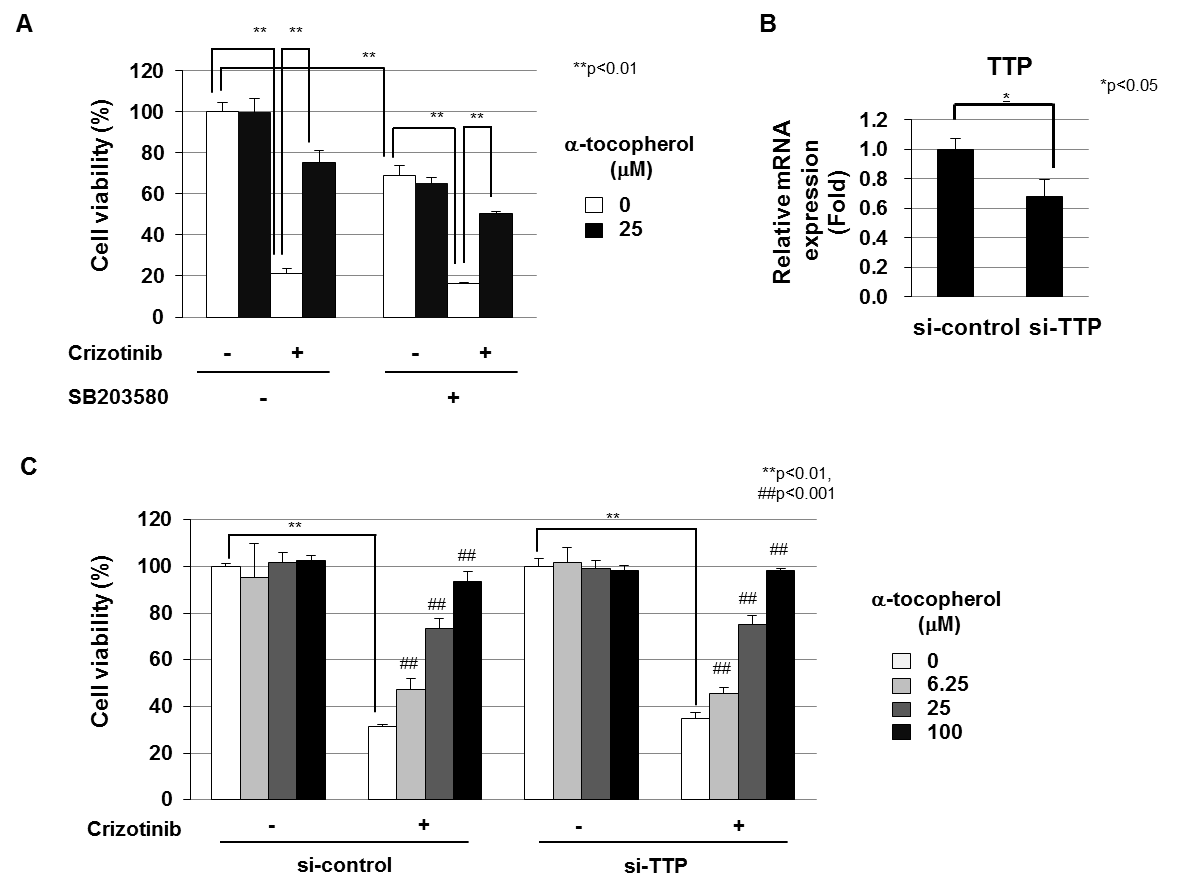
**

Supplement: S2 Fig — (A) Ba/F3 cells expressing NPM-ALK were treated with crizotinib (0.5 μM) in combination with α-tocopherol (25 μM) and/or SB203580 (30 μM) for 24 hr. Cell viabilities were measured by a WST assay. (B, C) Ba/F3 cells expressing NPM-ALK were transfected with control siRNA and siRNA against TTP (si-control, si-TTP). (B) After 48 hr, total RNA was extracted and RT was performed using an oligo (dT)20 primer. Quantitative real-time PCR was performed using an iCycler detection system (Bio-Rad, Berkeley, CA, USA). GAPDH mRNA was analyzed as an internal control. Values are the mean ± S.D. of three independent experiments. *P < 0.05 (C) After 48 hr, transfected cells were treated with crizotinib (0.5 μM) in combination with α-tocopherol (6.25, 25, 100 μM) for 24 hr. Cell viabilities were assessed by a WST assay. Values are given as the mean ± SD of four independent experiments. **P < 0.01. (DOCX) [file pone.0183003.s002.docx]

**
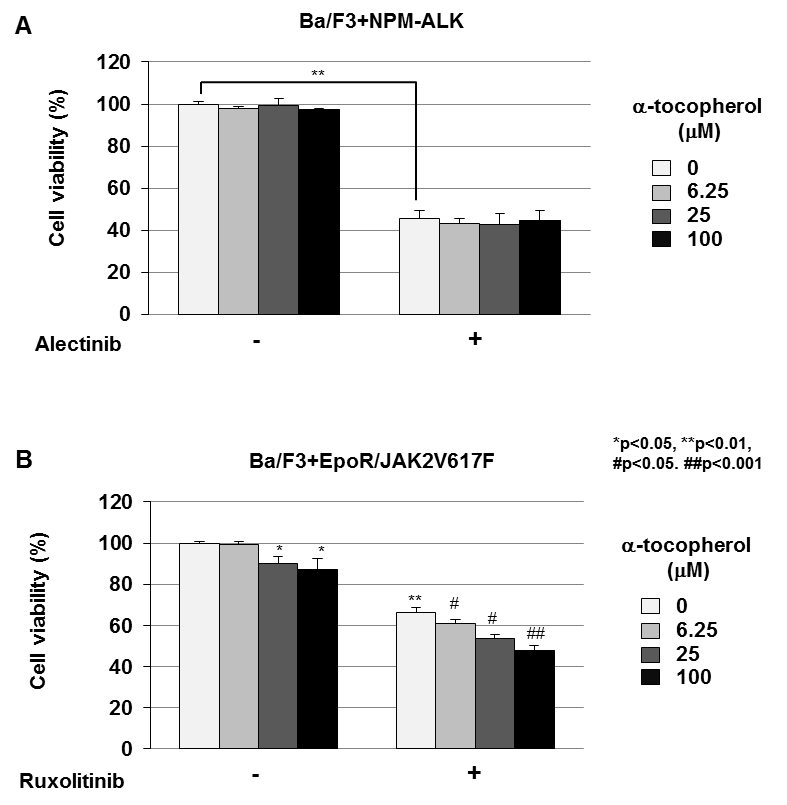
**

Supplement: S3 Fig — (A) Ba/F3 cells expressing NPM-ALK were treated with alectinib (0.1 μM) in combination with α-tocopherol (6.25, 25, and 100 μM) for 24 hr. Cell viabilities were evaluated by a WST assay. Values are given as the mean ± SD of four independent experiments. **P < 0.01 (B) Ba/F3 cells expressing the erythropoietin receptor (EpoR) and JAK2 V617F mutant were treated with ruxolitinib (0.3 μM) in combination with α-tocopherol (6.25, 25, 100 μM) for 24 hr. Cell viabilities were evaluated by a WST assay. Values are given as the mean ± SD of four independent experiments. **P < 0.01 significantly different from the control group; ##P < 0.01 significantly different from the group incubated with 0.3 μM ruxolitinib. (DOCX) [file pone.0183003.s003.docx]
